# Does a complex intervention increase patient knowledge about oral anticoagulation? - a cluster-randomised controlled trial

**DOI:** 10.1186/s12875-017-0588-2

**Published:** 2017-02-07

**Authors:** Verena Maikranz, Andrea Siebenhofer, Lisa-R. Ulrich, Karola Mergenthal, Sylvia Schulz-Rothe, Birgit Kemperdick, Sandra Rauck, Gudrun Pregartner, Andrea Berghold, Ferdinand M. Gerlach, Juliana J. Petersen

**Affiliations:** 1 Institute of General Practice, Goethe-University Frankfurt am Main, Frankfurt, Germany; 2 Institute of General Practice and Evidence-based Health Services Research, Medical University Graz, Auenbruggerplatz 2/9, A-8036 Graz, Austria; 3 Institute for Medical Informatics, Statistics and Documentation, Medical University Graz, Graz, Austria

**Keywords:** Oral anticoagulation, Patient education, Case management, General practice, Patient knowledge

## Abstract

**Background:**

Oral anticoagulation therapy (OAT) is a challenge in general practice, especially for high-risk groups such as the elderly. Insufficient patient knowledge about safety-relevant aspects of OAT is considered to be one of the main reasons for complications. The research question addressed in this manuscript is whether a complex intervention that includes practice-based case management, self-management of OAT and additional patient and practice team education improves patient knowledge about anticoagulation therapy compared to a control group of patients receiving usual care (as a secondary objective of the Primary Care Management for Optimised Antithrombotic Treatment (PICANT) trial).

**Methods:**

The cluster-randomised controlled PICANT trial was conducted in 52 general practices in Germany, between 2012 and 2015. Trial participants were patients with a long-term indication for oral anticoagulation. A questionnaire was used to assess knowledge at baseline, after 12, and after 24 months. The questionnaire consists of 13 items (with a range of 0 to 13 sum-score points) covering topics related to intervention. Differences in the development of patient knowledge between intervention and control groups compared to baseline were assessed for each follow-up by means of linear mixed-effects models.

**Results:**

Seven hundred thirty-six patients were included at baseline, of whom 95.4% continued to participate after 12 months, and 89.3% after 24 months. The average age of patients was 73.5 years (SD 9.4), and they mainly suffered from atrial fibrillation (81.1%). Patients in the intervention and control groups had similar knowledge about oral anticoagulation at baseline (5.6 (SD 2.3) in both groups). After 12 months, the improvement in the level of knowledge (compared to baseline) was significantly larger in the intervention group than in the control group (0.78 (SD 2.5) vs. 0.04 (SD 2.3); *p* = 0.0009). After 24 months, the difference between both groups was still statistically significant (0.6 (SD 2.6) vs. -0.3 (SD 2.3); *p* = 0.0001).

**Conclusion:**

Since this intervention was effective, it should be established in general practice as a means of improving patient knowledge about oral anticoagulation.

**Trial registration:**

Current controlled trials ISRCTN41847489; Date of registration: 13/04/2012

## Background

The prevalence of patients receiving anticoagulants in Germany is high, with about 930,000 people taking coumarins daily [1]. Adequate oral anticoagulation management is therefore a key challenge in the medical care of elderly patients in general practices [2]. In the general adult population in Germany, the 1-year prevalence of atrial fibrillation (AF), the most common indication for long-term oral anticoagulation [3], has recently been estimated at 2.3%, and it rises considerably with age [4]. Oral anticoagulation therapy (OAT) is also used for secondary prevention, e.g. in patients with recurrent thrombosis [5].

Vitamin K antagonists (VKA), which are still the oral anticoagulants that are most often prescribed in Germany [1], take fourth place in the List of High-Alert Medications consisting of drugs that are most likely to cause harm to patients [6]. The management of coumarin therapy is often challenging because the narrow therapeutic index (for patients with AF a target Internationalised Normalised Ratio (INR) of 2.0 to 3.0 is recommended) makes it difficult to achieve optimal doses of the medication, and food-drug interactions are common [7]. Improper handling can thus lead to dangerous bleeding complications or thromboembolic events. About 20% of hospitalisations in Europe as a result of acute stroke are related to atrial fibrillation [8].

The introduction of direct oral anticoagulants (DOACs) has had a considerable impact on the world of oral anticoagulation: In Germany, dabigatran, a direct thrombin inhibitor, and rivaroxaban, a factor Xa inhibitor, were the first DOACs to be approved for the prevention of stroke and systemic embolism in patients with non-valvular atrial fibrillation and at risk of stroke [9]. Since receiving approval in 2011, prescriptions of DOACs have risen strongly, with 38 million defined daily doses (DDDs) of DOACs prescribed in Germany in 2012 (vs. 389 mio. DDDs of VKAs), and 183 mio. DDDs prescribed in 2014 (vs. 364 mio. DDDs of VKAs) [10]. According to the GARFIELD registry (Global Anticoagulant Registry in the FIELD), which includes patients with newly diagnosed atrial fibrillation and at least one additional risk factor, the proportion of patients on DOACs in Germany increased from 4.3% in 2011 to 24.8% in 2014 [11]. Although DOACs are considered to be an effective treatment choice for long-term anticoagulation therapy, there have been a number of concerns [9]. For instance, the lack of a readily available monitoring test and the absence of specific antidotes (apart from dabigatran, for which an antidote was approved in 2015 [9]) are a problem in the event of DOAC-associated bleedings [12]. DOACs are not suitable for all patients: For instance, patients with mechanical heart valves should not use them [13]. Moreover, DOACs cost significantly more than VKA [10]: In Germany, the mean net cost of vitamin K antagonists is €0.18 per DDD, compared to €3.75 for dabigatran, and €3.45 for factor Xa antagonists.

Oral anticoagulation treatment by caregivers in clinical practice is often unsatisfactory. A systematic review indicates under-treatment of high-risk atrial fibrillation patients in two thirds of analysed studies [14]. Despite the need to maintain the INR within the guideline-recommended therapeutic target range in accordance with the indication [5], INR monitoring and documentation quality in German general practices have often been reported to be inadequate [15]. A cross-sectional survey by Chenot et al. indicates gaps in patient knowledge that reduce the safety and effectiveness of OAT. High age and low education levels are associated with lower overall safety-relevant knowledge about OAT [16, 17]. Insufficient patient knowledge about OAT is related to poorer anticoagulation control [18, 19]. An additional predictor of poor anticoagulation quality includes long intervals between measurements [20].

Patient education has been proposed as a means of improving adherence to oral anticoagulation [9, 21]. However, currently existing patient education strategies vary in terms of content, setting, duration, stated goal, structure and involved personnel [22]. In Germany, where general practitioners (GPs) manage the majority of patients on OAT [16], large-scale RCTs to test the effectiveness of clearly defined patient education interventions in primary care settings are needed [23]. To the best of our knowledge, no study has yet analysed the effectiveness of a complex intervention that includes major elements of well-established tools such as practice-based case management, self-management of OAT, and additional education for patients and practice teams to improve their knowledge about OAT. Previous education strategies that have successfully reduced thromboembolic complications and mortality rates have mostly been based on self-management of OAT [24–26]. Nonetheless, self-management may not be suitable for all patients [27]. Apart from self-management, case management, which involves systematically monitoring patients, encouragement to continue the treatment and action in the case of non-adherence or no improvement [28], has been successful in patients with other chronic conditions such as chronic heart failure [29], osteoarthritis [30], and depression [31].

Between 2012 and 2015 the Institute of General Practice, Goethe-University Frankfurt, Germany, conducted the cluster-randomised PICANT trial (Primary Care Management for Optimised Antithrombotic Treatment) on patients with a long-term (i.e., lifelong) indication for oral anticoagulants in general practices in Germany, with the primary aim of improving antithrombotic management in primary healthcare by reducing major thromboembolic and bleeding events requiring hospitalisation [32].

In this paper we investigate whether the complex PICANT intervention that includes practice-based case management, self-management of OAT and additional patient and practice team education leads to an increase in patient knowledge about anticoagulation therapy after 12 and 24 months in intervention recipients, compared to patients receiving usual care (as one of the PICANT trial’s secondary objectives).

## Methods

### Study design

The cluster-randomised controlled PICANT trial on anticoagulation management was conducted between June 2012 and March 2015 in 52 general practices in the federal states of Hesse and Rhineland-Palatinate. The study was approved on June 26th, 2012, by the ethical review committee of the University Hospital, Goethe-University Frankfurt, Germany. The study is registered at www.controlled-trials.com (ISRCTN41847489). The study protocol, the presentation of the Coagulation-Monitoring-List (Co-MoL), the practice and patient recruitment process and data on screened patients have already been published elsewhere ([32–34]). Publications are currently being prepared on the primary and secondary objectives.

### Study population

Patients of ≥ 18 years of age, with a long-term (lifelong) indication for oral anticoagulation (atrial fibrillation/flutter, recurrent venous thromboembolism or pulmonary embolism, mechanical heart prosthesis, and other conditions, such as hereditary coagulopathy, intracardial thrombosis) and who were taking any kind of OAT (coumarins, antiplatelet therapies, DOACs such as dabigatran) were recruited. Moreover, they had to regularly attend the GP’s practice and sign an information consent form. Exclusion criteria included dementia, a life expectancy of less than 6 months, drug or alcohol abuse, residence in a nursing home, or insufficient German language skills.

### Intervention

As described in the study protocol, the complex intervention included the provision of tools for study participants. These included information materials for patients, which depended on the type of oral anticoagulation they were taking, as well as information materials on oral anticoagulation and guidelines for GPs and healthcare assistants employed in their practices [32].

In Germany, GP practices generally employ one or more health care assistants (HCAs, ‘Medizinische Fachangestellte’). Their role is comparable to healthcare assistants in the UK or to medical assistants in the United States [35]. They perform basic clinical tasks such as intramuscular injections, ECGs, spirometry, and taking blood samples.

At a 1-day workshop, health care assistants were trained to perform case management and educate patients (including an information brochure and a video developed by Hua et al., both of which contained safety-relevant information relating to OAT) [36].

HCAs were taught to tell patients about their disease and treatment conditions, to encourage them to perform self-management and to monitor them regularly using the Coagulation-Monitoring-List (Co-MoL) explained in detail elsewhere [33]. The training also included DOAC-related topics (e.g. monitoring of renal function). HCAs conducted patient interviews following patients’ visits at the GP’s practice. In general, time intervals between monitoring visits depended on the stable adjustment of therapy. Most contacts took place in the practice, as the majority of anticoagulant patients visit the practice regularly anyway. If necessary, patients were contacted by telephone between the practice visits as well. The results of the interviews were reported to the GP who decided whether any further action was necessary. The aim was to assess symptoms and adherence to medication in patients, and to detect complications early and assess adverse effects.

GPs were provided with detailed explanations of what to expect from case management. Quality meetings were held three times during the course of the trial to discuss the newly approved DOACs, as well as practical problems involved in anticoagulation and individual case reports.

For patients, the complex intervention consisted of practice-based case management involving treatment monitoring, patient education, the provision of individual OAT-specific information, and encouragement to perform self-management where applicable. The control group received treatment as usual according to the current evidence-based guideline for oral anticoagulation therapy [37, 38].

### Data collection and patient knowledge questionnaire

The assessments occurred at baseline, after 12 and after 24 months. Patients completed a knowledge questionnaire developed by Hua et al. for a previous trial on education for patients receiving OAT [36, 39]. The questionnaire contains 13 items covering safety-relevant knowledge about OAT. It aims to measure what patients learned from the intervention. Five questions relate to the patient’s individual OAT therapy, such as the indication for oral anticoagulation and the expected duration of treatment. Four questions relate to drug and other interactions. The remaining four questions are about safety precautions, such as the recognition of complications or emergencies. Open-ended questions allow free-text answers.

After extracting the main information from the free-text answers given by patients, categories were constructed manually, and answers with similar content summarised. Key facts emphasised in the intervention were used to build the categories. Following the evaluation scheme of Vormfelde at al., each of the thirteen questions included in the questionnaire was weighted with one point. Wrong answers, replies such as “I don’t know”, missing answers, or answers that were not evaluable within the context of the question, received 0 points. The sum-score therefore ranged from 0 to a maximum of 13 points, with higher scores indicating greater knowledge about OAT [13]. The questionnaire was handed out to all patients (regardless of their OAT), because most questions were about general safety-relevant aspects of OAT. However, the evaluation scheme was modified to take into account particularities of DOACs, using current guidelines as a basis [21]. For instance, when asked about their individual monitoring intervals, patients who had switched to DOACs and gave answers such as “I no longer need monthly monitoring of coagulation in the general practice” were given one point in the corresponding item, whereas patients taking vitamin K antagonists were expected to report their individual monitoring intervals.

### Statistical analyses

Means and standard deviations of patients’ total sum-scores at baseline and at the two follow-ups were calculated for each group. Differences in the development of patient knowledge between the intervention and control groups compared to baseline were assessed separately for each follow-up assessment using linear mixed-effects models, and practices were included as random effects to account for the clustered structure of the data. A *p*-value of less than 5% was considered significant. SPSS version 19 and SAS version 9.4 were used for data analysis.

## Results

### GP and patient characteristics at baseline

The average age of the 52 general practitioners recruited for the trial was 50.9 (SD 7.7) years, and nearly two thirds of them were male (65.4%). The majority of the physicians were general practitioners (84.6%). Fewer than half of them (42.3%) worked in solo practices. In the intervention group, an average of 3 health care assistants worked in each practice, compared with a mean of 3.6 HCAs per practice in the control group.

Of the 1469 eligible patients who were invited to participate in the trial, 736 patients (50.1%) provided written informed consent and were included in the study at baseline [34]. Of these, 702 (95.4%) were still participating after 12 months, and 657 (89.3%) after 24 months. The remaining 733 patients (49.9%) did not participate. Participants and non-participants were comparable in age (73.5 (SD 9.4) vs. 75.0 years (SD 10.9)) and sex (male, 55.0% vs. 52.9%). A slightly higher percentage of participants performed self-management compared to non-participants (11.6% vs. 7.0%).

The median age of the participants was 73.5 (SD 9.4) and 55.0% were male (56.2% in the intervention group vs. 53.9% in the control group). Atrial fibrillation was the most common indication for anticoagulation (81.1%), followed by recurrent venous thrombosis (9.8%), other indications (9.1%) and recurrent pulmonary embolism (8.3%). At baseline, most patients received coumarins (94.3%), while 4.9% of all patients took DOACs. The last measured INR value lay within the therapeutic target range in approximately two thirds of patients (65.1%). A minority of patients (11.5%) performed self-management (self-testing and dose-adjusting). According to the GPs, most patients (80.0%) showed “very good compliance”. Baseline characteristics of the control and intervention arm are shown in detail in Table 1. Groups were comparable in terms of sex, age, indication for oral anticoagulation therapy, and type of medication.

**Table 1:** Sociodemographic and clinical characteristics of the study population at baseline

Patient characteristics	Intervention group	Control group	Total
	(*n* = 365)	(*n* = 371)	(*n* = 736)
Age (years), mean (SD)	74.4 (9.5)	72.8 (9.3)	73.5 (9.4)
Sex, n (%)			
Male	205 (56.2)	200 (53.9)	405 (55.0)
Female	160 (43.8)	171 (46.1)	331 (45.0)
CHA_2_DS_2_-VASc score^a^			
= 1, n (%)	9 (3.0)	12 (4.1)	21 (3.5)
> 1, n (%)	292 (97.0)	282 (95.9)	574 (96.5)
Long-term indication for oral anticoagulation, n (%)			
Atrial fibrillation	302 (82.7)	295 (79.5)	597 (81.1)
Recurrent venous thrombosis	32 (8.8)	40 (10.8)	72 (9.8)
Recurrent pulmonary embolism	31 (8.5)	30 (8.1)	61 (8.3)
Mechanical heart prosthesis	29 (7.9)	28 (7.5)	57 (7.7)
Intracardiac thrombus	3 (0.8)	4 (1.1)	7 (1.0)
Other indications	33 (9.0)	34 (9.2)	67 (9.1)
Antithrombotic medication, n (%)			
Coumarin derivates	346 (94.8)	348 (93.8)	694 (94.3)
Dabigatran	10 (2.7)	3 (0.8)	13 (1.8)
Rivaroxaban	7 (1.9)	16 (4.3)	23 (3.1)
Other	2 (0.5)	4 (1.1)	6 (0.8)
Last INR measured within therapeutic target range, n (%)	240 (65.8)	239 (64.4)	479 (65.1)
INR self-management, n (%)	39 (10.7)	46 (12.4)	85 (11.5)
Migration background^b^, n (%)	27 (7.4)	24 (6.5)	51 (6.9)
Patient compliance^c^, n (%)			
Very good compliance	308 (84.4)	266 (71.7)	574 (80.0)
Good compliance	51 (14.0)	86 (23.2)	137 (18.6)
Non-compliant	6 (1.6)	17 (4.6)	23 (3.1)
No assessment available	0	2 (0.5)	2 (0.3)

^a^Based on 595 patients with atrial fibrillation (301 in the intervention group and 294 in the control group), whose score data was available

^b^The population group with a migration background consists of all persons who have immigrated to the territory of today’s Federal Republic of Germany since 1949, all foreigners born in Germany, and all persons born in Germany who have at least one parent who immigrated to the country or was born as a foreigner in Germany (source: Federal Statistical Office)

^c^As assessed by GP

### Patient knowledge about oral anticoagulation at baseline

**Fig. 1:**
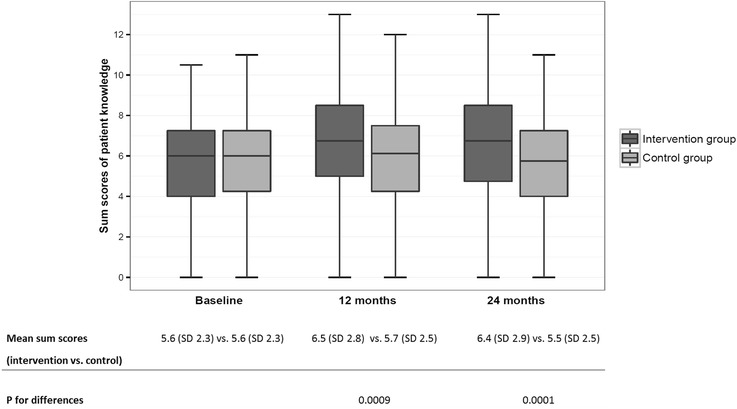
Comparison of patients’ level of knowledge about OAT between intervention and control groups

At baseline, intervention and control patients presented similar mean values for their knowledge about OAT (5.6 (SD 2.3) vs. 5.6 (SD 2.3)) (Fig. 1). The maximum number of points achieved at baseline was 10.5 in the intervention vs. 11.0 in the control group. About 60% knew their indication for OAT (question 1, see Table 2). At least 2/3 of all participants were aware of the goal of oral anticoagulation therapy (question 2) and their individual required treatment duration (question 3). Major knowledge gaps were observed in specific and detailed knowledge of OAT. About one third of patients knew their personal INR target range (VKA patients), or that they did not have an INR target range (DOAC patients) (question 5). 88.2% of all patients did not know about analgesics that are safe for orally anticoagulated patients and available without a prescription (question 8). 17.9% of participants were aware of the precautions that should be taken after forgetting to take a dose (question 9). 6.5% of participants were aware of emergency situations, such as melaena and impaired vision, that require a doctor’s visit the same day (question 12).

**Table 2:** Patients’ safety-relevant knowledge of OAT: Proportion of patients giving correct answers (at a single-item level)^a^

Item no.	Item	Intervention group			Control group		
		Baseline	After 12 mo.	After 24 mo.	Baseline	After 12 mo.	After 24 mo.
1	Indication for oral anticoagulation	60.0%	61.3%	60.8%	60.9%	57.3%	55.7%
2	Awareness of risk treated with OAT	67.7%	67.9%	65.4%	70.6%	67.4%	64.9%
3	Duration of treatment known	70.7%	74.9%	76.8%	68.7%	69.1%	68.6%
4	Checking frequencies known	37.8%	46.5%	44.0%	36.7%	42.4%	41.2%
5	Target INR range known	37.8%	59.5%	56.9%	36.1%	45.5%	43.7%
6	Foods which contain a large amount of vitamin K	29.0%	34.1%	31.6%	30.2%	29.2%	25.3%
7	Diet-related recommendations	70.7%	68.2%	69.3%	65.5%	67.4%	66.8%
8	Safest analgesic that is available without a prescription	11.0%	25.1%	26.8%	12.7%	15.4%	13.8%
9	What to do after missing medication dose	19.5%	28.6%	30.1%	16.4%	19.9%	16.3%
10	Awareness that underdosing results in no symptoms	14.5%	15.6%	15.7%	13.7%	14.6%	10.2%
11	Interactions with OAT	20.0%	26.9%	28.3%	19.4%	22.8%	20.3%
12	Recognition of emergencies (doctor’s visit necessary)	6.3%	22.5%	16.3%	6.7%	5.9%	6.8%
13	Knowing when it is important to inform others of OAT	26.0%	35.0%	30.4%	27.8%	23.9%	19.1%

^a^Analyses are based on 736 patients at baseline, 702 after 12 months and 657 patients after 24 months. 12 and 24-month follow-up data for the secondary endpoint patient knowledge were available for all patients who did not drop out. After 24 months, 79 patients (10.7%) dropped out because of death or the patient’s decision to no longer participate

### Patient knowledge after 12 months

After 12 months, participants in the intervention group showed higher knowledge values than those in the control group (6.5 (SD 2.8) vs. 5.7 (SD 2.5)) (Fig. 1). Compared to baseline, the number of correctly answered questions increased by 0.78 (SD 2.5) in the intervention group as compared to 0.04 (SD 2.3) in the control group (*p* = 0.0009). In general, scores on questions that were answered well before the intervention (see above) showed less improvement than scores on questions that were answered poorly (see Table 2). At 12 months, a greater percentage of intervention recipients than control recipients knew about the recommended non-prescription analgesic for patients with OAT (question 8) (25.1% vs. 15.4%). Members of the intervention group scored better than control group members with respect to measures to be taken after forgetting a dose (question 9) (28.6% vs. 19.9%). Intervention recipients had greater knowledge of emergency situations such as strokes and bleeding complications than members of the control group (question 12) (22.5% v. 5.9%).

### Patient knowledge after 24 months

At 24 months, the intervention group still returned higher scores than the control group (6.4 (SD 2.9) vs. 5.5 (SD 2.5)) (Fig. 1). Overall, the average scores were slightly lower in both groups than 12 months before. Compared to baseline, the level of knowledge of intervention recipients remained high, whereas patients in the control group showed a slight decline (0.6 (SD 2.6) vs. -0.3 (SD 2.3); *p* = 0.0001). The positive effect of patient education had not increased further at 24 months but remained approximately the same.

## Discussion

We present the results of the cluster-randomised controlled Primary Care Management for Optimised Antithrombotic Treatment study assessing whether patients with a long-term indication for oral anticoagulation knew more about OAT following a structured complex intervention. The complex intervention included practice-based case management, self-management of OAT and additional education for patients and practice teams. Compared to baseline, intervention recipients achieved a greater improvement in knowledge than patients in the control group after both 12 and 24 months. On a single item level, intervention patients made particular progress on items relating to the INR target range, the knowledge of a safe over-the-counter analgesic, precautions to be taken after forgetting a dose and the recognition of emergency situations.

### The meaning of the results in the context of the literature

Although a large number of patients are treated with oral anticoagulants in German general practices, no standardised patient education strategy exists [22]. Patients at risk of poor anticoagulation control (among others, patients with long intervals between measurements) are more likely to need therapy support through additional training [20]. Moreover, socio-demographic factors such as high age can negatively impact knowledge and understanding of oral anticoagulation, and this should be taken into consideration when developing education programmes [17]. Previous studies have demonstrated the need for ongoing OAT education, and periodic “refreshers” have been recommended for patients in order to maintain a satisfactory level of patient knowledge [40]. Strategies used to provide patient education on OAT cover a wide spectrum that includes the use of written information materials either alone or in addition to self-management interventions [41]. Written information materials may be most effective when they include simple and easy-to-understand information that takes readability into account [17]. Although self-management has proved to be successful in reducing thromboembolic complications and mortality rates by half [26], it is not feasible for all patients [27]. To our knowledge this is the first randomised controlled trial that has tested whether a complex patient education intervention that consists of practice-based case management and additional patient education can effectively raise knowledge that is relevant to patient safety.

Our findings are similar to those found in other recently conducted prospective studies [42, 43] and RCTs [44, 45] that describe an increase in knowledge resulting from patient education delivered face-to-face and by means of written information materials on OAT (without self-management). Furthermore, one prospective study showed that education that is focused mainly on the self-management of OAT can lead to an increase in patient knowledge [46]. However, most previous interventions were conducted in hospitals [42, 44, 45], teaching centres [46] and anticoagulation clinics [43], whereas our intervention took place in general practices, where the majority of patients on OAT are treated in Germany [2]. Most previous studies were characterised by small sample populations of fewer than 100 patients [42, 44–46]. In contrast, our trial included 736 participants at baseline. Moreover, most trials only analysed the short-term effects of educational programmes by assessing knowledge directly after the intervention [43, 44], whereas we were able to show that a high knowledge level was maintained in the intervention group over a period of 2 years. After 24 months, the average measures of knowledge had declined slightly in both groups but still remained at a high level among intervention recipients. Similar developments were observed in other trials [42, 46].

Our results are consistent with the German RCT by Vormfelde et al. which used the same questionnaire to evaluate patient knowledge after a patient education intervention [39]. However, the interventions were different. In Vormfelde’s trial, HCAs provided patients with verbal information during training sessions, as opposed to our case management with additional patient education [39]. In both trials, substantial gaps in patients’ knowledge were revealed at baseline [39]: It is worrying that less than half of the patients on VKA in both trials were aware of their personal INR target range. In both trials, fewer than 25% of patients knew the safest over-the-counter drug for patients on OAT. In both studies, knowledge of potential emergency situations was low. However, the results in our trial were even worse, since less than 10% of the baseline population were able to recognise symptoms of stroke or bleeding complications. Our results are similar to other studies that identified inadequate patient knowledge about oral anticoagulants [47, 48].

Higher test scores in the intervention group at both follow-up time points confirmed the effectiveness of the interventions in both trials. However, in comparison to Vormfelde et al. the study population in the PICANT trial was more than twice as large (736 patients vs. 319 patients) which implies greater power to detect differences between intervention and control groups [49].

This study took place against a background of increasing prescriptions of DOACs. Approved in 2011, dabigatran and rivaroxaban were the first DOACs to be approved for the prevention of stroke and systemic embolism. They were approved shortly before we submitted the study protocol to the ethics committee in November 2011. Nevertheless, we addressed DOAC-related issues in our intervention, e.g. by providing study participants with specific DOAC-related information materials. Our trial was representative for the uptake patterns of DOACs in Germany at the start of patient recruitment: According to the GARFIELD-AF registry, the proportion of patients on DOACs in Germany was 4.3% in 2011 [11]. Similar results were observed for our study population at baseline (in 2012, 4.9% of patients were taking DOACs). The number of patients on DOACs has increased strongly during the last 5 years [50]. This seems to be especially true for patients with newly diagnosed atrial fibrillation [51]. The percentage of VKA patients switching to DOACs was 7.6% in this trial, slightly lower than the results of a study by Bleckwenn et al., which was also conducted in German primary care practices [51].

For decades, the traditional concept in Germany has been one of “the-doctor-does-it-all” [35]. However, as a steadily decreasing number of GPs (especially in rural areas) are facing an increasing number of patients with complex needs, the role of healthcare assistants is being expanded to include not only administrative and simple tasks but also more complex ones, such as managing patients with chronic heart failure [29], osteoarthritis [30], and depression [31]. Since registered nurses do not work in German primary care, expanding the role of health care assistants to permit them to take greater responsibility provides important benefits for GPs [35]. The complex intervention described here was designed to be provided in addition to standard care of orally anticoagulated patients in general practices. It can be implemented in everyday care without the need to employ additional staff. Furthermore, recently developed reimbursement schemes in primary care (e.g. GP-centred care contracts) increasingly reimburse extra spending on specifically trained healthcare assistants and this is likely to further expand their role [35, 52].

Team-based care and task substitution help to maintain quality of care delivery in the face of growing demand for care among ageing populations, the increased prevalence of chronic diseases, and a shortage of GPs (particularly in rural areas of Germany). According to GPs, task shifting improves cooperation and information sharing in practice teams [53].

### Strengths and limitations

To the best of our knowledge, PICANT is the largest cluster-randomised controlled trial to have been conducted on the effect of a complex intervention that includes practice-based case management, self-management of OAT and additional patient education on therapy-related knowledge about oral anticoagulation in Germany. In addition to the large sample size, the strengths of the trial also include the long-term intervention period of 24 months. With the support of GPs and HCAs, the intervention is feasible in a ‘real world’ setting and does not require new personnel or the creation of new interfaces. According to the literature, team-based care involving HCAs is associated with greater patient satisfaction [54] and possibly with greater patient compliance as well. A trusting relationship to the care-provider is considered to be an important criterion in patient-oriented case management [55]. In this trial, patients on DOACs were also taken into account since there is a need for an analogous education programme, as explained above.

We acknowledge a potential selection bias, since a higher percentage of participants performed self-management compared to non-participants (11.6% vs. 7.0%). Participants may therefore have been more motivated than the eligible population from which we drew the sample. However, in terms of age and sex, no relevant differences between participants and non-participants could be identified. Up to now, the effectiveness of the complex intervention has only been evaluated in terms of whether it leads to an increase in patient knowledge about oral anticoagulation. We have not yet analysed the relationship between greater knowledge and improved clinical outcomes such as bleeding and thromboembolic complications, as well as time in the INR target range. Since the intervention is complex, evaluations stemming from qualitative interviews with participants need to be assessed in order to completely understand the mechanism of action. We have not referred specifically to the Medical Research Council guidance for the evaluation of complex interventions [56] since we did not strictly follow all the guideline’s recommendations on process evaluation. However, key guideline recommendations were taken into account in the development, evaluation and implementation of the intervention.

A notable loss to follow-up occurred at 24 months, which is a common problem in primary care trials (79 of 736 patients, 9.0% in the intervention group vs. 12.4% in the control group). The reasons for this were death or the patient’s decision not to participate any further. Vormfelde and colleagues reported that 95.4% of patients in the intervention group completed the 6-month follow-up assessment, compared with 88.7% in the control group [39]. Nonetheless, our analyses reveal that the impact of the intervention on patient knowledge remained statistically significant, despite missing data resulting from nonparticipation in follow-up assessments. Our educational training intervention and the cluster design of the trial did not permit the practice team, patients and researchers to be blinded to group assignment.

A further limitation of this study is that we used a patient questionnaire on OAT knowledge that was originally developed for VKA patients [36]. It would have been difficult to distribute different questionnaires for patients taking different oral anticoagulants (VKA and DOACs) because a DOAC knowledge questionnaire of comparable quality was not available in 2011, and because a relatively high proportion of patients switched therapy in the course of the trial (mainly from VKA to DOACs, but also from DOACs to VKA). For future research projects, however, we recommend revising the questionnaire by creating three modules (one common module on general safety, one module with VKA-specific questions and one module specific to DOACs). The need for education remains, even when vitamin K antagonists are replaced by direct oral anticoagulants. Since a readily available monitoring test does not exist for DOACs and dosing depends on renal function, patients should therefore be informed about safety-relevant aspects of their anticoagulant therapy. Amara et al. have recently shown that there are major knowledge gaps among DOAC patients [57]: Only 53.9% of all DOAC patients were aware of the fact that regular monitoring of renal function is recommended for patients taking DOACs.

## Conclusion

Our study showed that patient knowledge of INR target ranges, the proper use of medication to avoid drug interactions (e.g. with over-the-counter analgesics), and the recognition of emergencies such as strokes or bleeding complications, is often lacking. A complex intervention including general practice-based case management, self-management of OAT and additional education for patients and practice teams can lead to growth in patient knowledge. Since this intervention is only dependent upon the involvement of the entire general practice team and requires no additional interface, it would be possible to establish it as a measure to improve oral anticoagulation management in the German primary care sector. The need for education remains, even when vitamin K antagonists are replaced by direct oral anticoagulants. Since a readily available monitoring test does not exist for DOACs, and dosing depends on renal function, patients should be informed about safety-relevant aspects of their anticoagulant therapy. Educational programmes should contain standardised, clearly defined content focusing particularly on the safety-relevant gaps in knowledge that were identified in the baseline population. Patient education needs to be comprehensive and comprehensible, especially for older patients. Moreover, it can be presumed that patients should be educated more than once if a satisfactory level of patient knowledge is to be maintained.

## Abbreviations

AF: Atrial fibrillation
Co-MoL: Coagulation-Monitoring-List
DDDs: Daily Defined Doses
DOACs: Direct oral anticoagulants
GARFIELD: Global Anticoagulant Registry in the FIELD
GPs: General practitioners
HCA: Health care assistant
INR: International Normalised Ratio
OAT: Oral anticoagulation therapy
PICANT: Primary Care Management for Optimised Antithrombotic Treatment
RCT: Randomised controlled trial
VKA: Vitamin K antagonists
